# Identification and characterization of abundant repetitive sequences in *Eragrostis tef* cv. Enatite genome

**DOI:** 10.1186/s12870-016-0725-4

**Published:** 2016-02-01

**Authors:** Yohannes Gedamu Gebre, Edoardo Bertolini, Mario Enrico Pè, Andrea Zuccolo

**Affiliations:** Institute of Life Sciences, Scuola Superiore Sant’Anna, Piazza Martiri della Libertà, 33-56127 Pisa, Italy; Department of Dryland Crop and Horticultural Sciences, College of Dryland Agriculture and Natural Resources, Mekelle University, P.O.Box 231, Mekelle, Ethiopia

**Keywords:** Eragrostis tef, Repetitive sequences, Transposable Elements, Satellite sequences

## Abstract

**Background:**

*Eragrostis tef* is an allotetraploid (2n = 4 × = 40) annual, C4 grass with an estimated nuclear genome size of 730 Mbp. It is widely grown in Ethiopia, where it provides basic nutrition for more than half of the population.

Although a draft assembly of the *E. tef* genome was made available in 2014, characterization of the repetitive portion of the *E. tef* genome has not been a subject of a detailed analysis.

Repetitive sequences constitute most of the DNA in eukaryotic genomes. Transposable elements are usually the most abundant repetitive component in plant genomes. They contribute to genome size variation, cause mutations, can result in chromosomal rearrangements, and influence gene regulation. An extensive and in depth characterization of the repetitive component is essential in understanding the evolution and function of the genome.

**Results:**

Using new paired-end sequence data and a de novo repeat identification strategy, we identified the most repetitive elements in the *E. tef* genome. Putative repeat sequences were annotated based on similarity to known repeat groups in other grasses.

Altogether we identified 1,389 medium/highly repetitive sequences that collectively represent about 27 % of the teff genome. Phylogenetic analyses of the most important classes of TEs were carried out in a comparative framework including paralog elements from rice and maize. Finally, an abundant tandem repeat accounting for more than 4 % of the whole genome was identified and partially characterized.

**Conclusions:**

Analyzing a large sample of randomly sheared reads we obtained a library of the repetitive sequences of *E. tef*. The approach we used was designed to avoid underestimation of repeat contribution; such underestimation is characteristic of whole genome assembly projects. The data collected represent a valuable resource for further analysis of the genome of this important orphan crop.

**Electronic supplementary material:**

The online version of this article (doi:10.1186/s12870-016-0725-4) contains supplementary material, which is available to authorized users.

## Background

Eukaryote genomes show a striking variation in size. The variation does not correlate with the biological complexity of the organisms; indeed, gene content remains quite similar across different species. This phenomenon has been described as the “*C*-value paradox” where the 1C DNA value is the quantity of DNA in a gamete [1]. Genome size variation is extremely evident in plants spanning at least three orders of magnitude between the 1C DNA content genome of *Genslisea margaretae* (58.68 Mb) [2] and the 1C DNA content of *Paris japonica* (148,648 Mb) [3]. Interestingly, polyploidy accounts for very little of the “*C*-value paradox.” The majority of variation in plant genome sizes is based on differences in repeat sequence content [4].

Repetitive sequences include: tandem-arranged satellite sequences, telomeric sequences, microsatellite sequences, ribosomal genes, and transposable elements (TEs) [5]. TEs, also known as transposons or mobile elements, are DNA sequences ubiquitously found in almost all living organisms and capable of replication and movement to different parts of the host genome [6]. Depending on the mechanism adopted during transposition and/or to the molecule used as an intermediate, they are hierarchically classified to two major classes: Class I, (or RNA transposons or retrotransposons) and Class II (DNA transposons). Class I TEs use RNA as an intermediate molecule for replication and move through a “copy and paste” mechanism. On the other hand, Class II elements do not exploit an RNA intermediate and use a “cut and paste” mechanism to move [7, 8].

TEs are interspersed across the genome and largely contribute to plant genome size variations. For instance, the overall TE contents in different rice species vary from 25 % to 66 % [9]. TE content is 61 % in sorghum [10], more than 85 % in maize [11], and 95 % in bread wheat [12]. TEs amounts can differ quite dramatically between closely related organisms. A striking example is *Oryza australiensis* which has nearly doubled its genome size due to repeat amplification in less than three million years of evolution [13].

The movement and amplification of TEs can cause mutations [14], produce chromosomal rearrangements [15], affect gene regulation [16, 17] and promote exon shuffling [18, 19]. TE sequences can be co-opted by the host genome, in a process called exaptation, acquiring new and potentially beneficial functions [20, 21]. TEs are also amenable tools in phylogenetic and population studies [22], where they are used as a source of genetic markers [23–25]. Because of the deleterious effects that TE amplification can have on host genomes, these elements are normally under tight control. Indeed the majority of TEs are inactivated or silenced by mutation or epigenetic mechanisms including DNA and histone methylation as well as small interfering RNA (siRNA) activity [26, 27]. Plants counteract genome expansion due to TE amplification mostly by two mechanisms leading to the partial removal of TE related sequences: unequal recombination and illegitimate recombination [28, 29].

The presence of TEs complicates the genome assembly process [30] and leads to difficulties in gene annotation [31]. The identification of repetitive DNA has thus become an essential part of genome annotation [22].

Our research focuses on the characterization of the repetitive fraction of teff (*Eragrostis tef)* cv Enatite genome. The genus *Eragrostis* is part of the grass family Poaceae (Gramineae) [32] and contains 350 species, of which about 69 % are characterized by polyploidy, ranging from diploids (2n = 2 × = 20) to hexaploids (2n = 6×= 60) [33]. *E. tef* is an allotetraploid (2n = 4 × = 40) with an estimated nuclear genome size of 730 Mbp [34], which is roughly the same size as diploid sorghum and about 60 % larger than the diploid rice genome. *E. tef is* a C4 annual grass [35] which is widely grown and well adapted in Ethiopia, where it provides basic nutrition for more than half of the population [36]. However there are many constraints such as low productivity and lodging [37, 38] that still affect teff production and need to be addressed to improve total yield.

A draft assembly of *E. tef* genome was released in 2014 [36]. However compared to other major cereals many genomic features of *E. tef* remain poorly characterized. In particular the repetitive component has only been marginally investigated to date.

In order to collect a representative sample of the medium/highly repetitive fraction of tef genome, a de novo identification strategy was adopted to analyze a large dataset of random sheared reads. Similarity and structural feature searches were then carried out to gain a better insight into the repetitive component. A library composed of 1,389 different medium/high repetitive sequences was isolated. Altogether the library is representative of about 27 % of the teff genome. Phylogenetic analyses were carried out to study the most important TE classes in a comparative framework using TE paralogs from rice and maize. We identified and partially characterized an abundant tandem repeat that accounts for more than 4 % of the whole teff genome.

## Results

Half a million paired-end reads representing 0.25× coverage of the *E. tef* genome were analyzed using RepeatScout [39], a program that has proven effective in de novo identification of repeats. Reads were assembled into consensus sequences using CAP3 [40], and consensus sequences were clustered into repeat groups using cd-hit [41]. Altogether, the two sets total 184,986 bp which corresponds to ~0.25 × coverage of the estimated *E. tef* genome (i.e. 730 Mbp). This coverage of the genome is greater than those used in several low-pass sequencing analyses which have been used to capture and characterize the medium/highly repetitive fraction of a genome [42–44].

### Repeats library-composition and characterization

A set of 1,389 different medium/highly repetitive sequences (library Etef_repeats_V1.4) (Additional file 1) were identified in the *E. tef* genome. Similarity searches and structural feature analyses were used to better characterize these sequences. The most represented TE class in the repetitive library was that of Long Terminal Repeat Retroelements (LTR-RT) accounting for 31.82 % of the entries. In particular, Ty1-copia and Ty3-gypsy elements represented 12.17 % and 16.99 % of the library, respectively. A small amount (2.66 %) of the LTR-RT sequences identified were not convincingly associated with either of the two superfamilies. Another 1.80 % of the isolated repetitive sequences shared similarity with non-LTR retroelements. Class II DNA element sequences represented 9.14 % of the repetitive library. SINEs only accounted for 0.5 % representation in the repetitive dataset. Roughly 1 % of the sequences were associated with other classes of TEs or repetitive sequences. Finally, 55.51 % repetitive sequences were not clearly associated with any TE class on the basis of similarity searches (Table 1).

**Table 1:** Repeat library composition and abundance estimate

Repeat class		Number of sequences in rpt library	Estimated abundance in genome (%)
Class I	LTR-RT (all)	442	14.96
	Ty1-copia	169	2.67
	Ty3-gypsy	236	11.40
	Unclassified LTR-RT	37	0.89
	Non-LTR retroelements	25	0.12
	SINEs	7	0.18
Class II	DNA TE (including MITEs)	127	2.33
	Other TEs	14	0.89
	Satellite	3	4.54
	Uncharacterized	771	4.44
	Total	1389	27.46

In order to calculate the relative abundance of different repeats in the *E. tef* genome, a subset of 250,000 random sheared sequences with an average length of 367 bp was searched using RepeatMasker [45] with the Etef_repeats_V1.4 library used as a reference. Altogether the Etef_repeats_V1.4 library masked 27.46 % of the random sheared sequence set. The most represented TE class was LTR-RTs, totaling 14.96 %. Ty3-*gypsy* superfamily was more abundant than Ty1-*copia*: 11.40 % vs. 2.67 %. Repeats similar to LTR-RTs but not classifiable into either of the two subfamilies masked 0.89 % of the dataset. Non-LTR retrotransposons account for 0.12 %, a value similar to those observed in many plant genomes. Class II DNA elements, including MITEs, accounted for 2.33 % of the genome. A single repetitive sequence alone seemed to be present in a great copy number in the teff genome, covering 4.54 % of the sampled sequence set. When the three copies of this sequence present in the library were analyzed for structural features using dot plot comparison and Tandem Repeats Finder [46], a tandem arrangement was clearly recognized (Additional file 2). We further tested this hypothesis in order to better characterize this sequence (see the subsection: An abundant Satellite sequence).

### Assessing the completeness of the library

The Etef_repeats_V1.4 library was compared to libraries generated from random *E. tef* reads using the tools RepArk [47], TeDNA [48], and RepeatExplorer [49]. When the Etef_repeats_V1.4 library was used to mask the 1,091 repetitive sequences isolated by RepArk, it masked 56.54 % of the total number of candidates. Through similarity searches, the remaining 43.46 % of sequences were characterized as plastidial, ribosomal, and bacterial contaminants. On the other hand, RepArk candidates masked just 29.33 % of the Etef_repeats_V1.4 repetitive library. Consequently, it appears that RepArk missed most of the repeats without capturing any new ones. Similarly, in the same analysis carried out on the TeDNA output (306 sequences), Etef_repeats_V1.4 masked 55.83 % of TeDNA candidates, the remaining ones being plastidial contaminants. TeDNA output masked only 29.55 % of Etef_repeats_V1.4. Finally, Etef_repeats_V1.4 masked 87.11 % of the 2,722 sequences belonging to the two hundred most abundant clusters identified by Repeat Explorer. The unmasked candidates were represented by plastidial sequences, tracts of gene families, and other contaminants. RepeatExplorer library masked 78.24 % of Etef_repeats_V1.4. Altogether these data suggest that the library Etef_repeats_V1.4 is highly representative, i.e., RepeatScout was able to collect most repeats from a given dataset (Table 1).

### Phylogenetic analyses

Paralogs tracts from the reverse transcriptase (RT) coding domains of LTR-RTs and non-LTR retroelements were retrieved from a subsample of 250,000 random sheared *E. tef* sequences. Paralog elements from the most abundant and studied LTR-RT elements in maize and rice were mined from the public database MaizeDB (http://maizetedb.org/~maize/), Retroryza [50] and Repbase [51].

The data collected were then aligned (Additional files 3, 4 and 5) and used to build phylogenetic trees using the neighbor-joining (NJ) method and calculating the bootstrap values for 1,000 replicates.

**Fig. 1:**
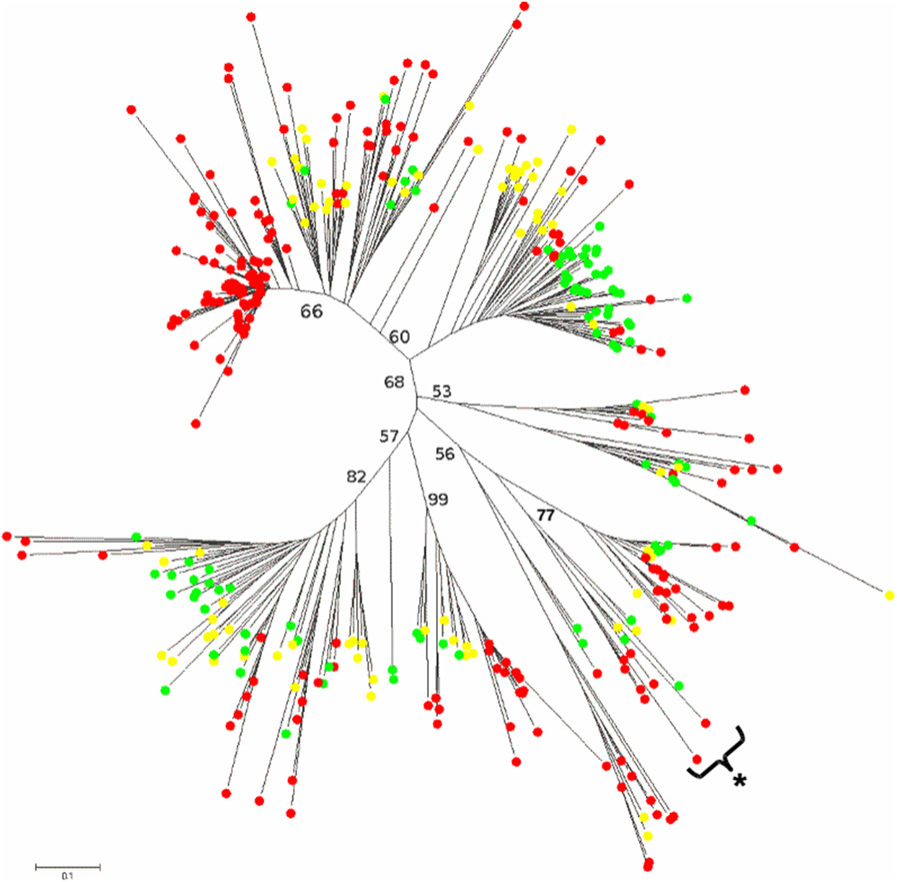
Phylogenetic analysis of Ty1-***copia*** retroelements**.** Bootstrap values were calculated for 1000 replicates; only those greater than 50 are shown. Paralogs from maize elements are marked with yellow circles; those from rice with green circles, and those from teff with red circles. “*” indicates the clade containing elements related to the rice LTR-RT family RIRE1

In the case of Ty1-*copia* elements, 385 paralogs tracts were analyzed: 215 from teff, 93 from rice, and 77 from maize (Fig. 1).

Under the assumption that *Zea* and *Oryza* genera diverged 55 million years ago (mya) [52, 53] the phylogenetic distance separating *Zea* and *Eragrostis* genera was estimated at 36.47 (20.64–50.54) mya [54].

In most of the bootstrap supported clades, the elements from the three different species mixed together. There was however, a single clade with high bootstrap support including 85 teff paralogs (39.5 % of the total amount of tracts used), possibly representing a teff specific Ty1-copia family.

**Fig. 2:**
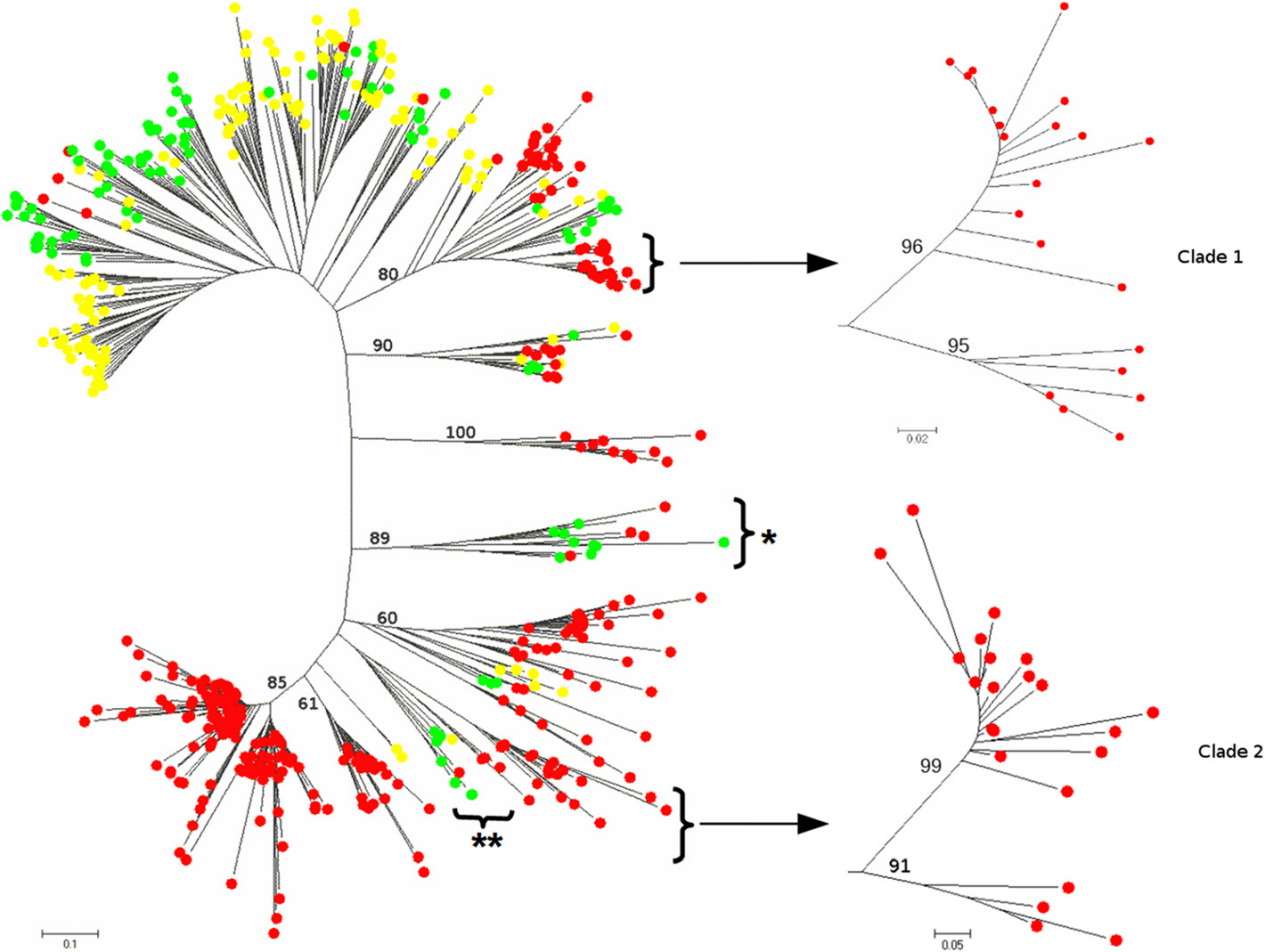
Phylogenetic analysis of Ty3-gypsy retroelements. Bootstrap values were calculated for 1000 replicates; only those greater than 50 are shown. Paralogs from maize elements are marked with yellow circles; those from rice with green circles and those from teff with red circles. “*” indicates the clade related to the rice LTR-RT family Atlantys. “**” indicates the clade related to the rice LTR-RT family RIRE2. The details of two clades splitting into two subclades are presented on the right (and in Additional files 6, 7 and 8)

In the case of Ty3-*gypsy* elements, 515 paralogs were analyzed: 295 from teff, 97 from rice, and 123 from maize. This scenario is quite different from the one described for Ty1-copia with most of the teff Ty3-*gypsy* paralogs collapsing in species-specific clades. A single teff specific clade alone included 162 paralogs out of the 295 used for this species (54.9 %). Mixed clades on the other hand comprised only a minor fraction of the paralogs. The clades containing the highly abundant *Oryza sativa* Ty3-gypsy elements Atlantys [55] and RIRE2 [56] as well as those containing elements of the abundant Ty1-copia family RIRE1 [13], included only a limited amount of *E. tef* paralogs, thus indicating that the elements related to these families are present but not abundant in teff. In the Ty3-gypsy NJ tree two teff specific clades were identified, each containing two separate subclades both with high bootstrap support (Fig. 2). These are the only clades showing such features that were identified in both Ty1-copia and Ty3-gypsy the NJ tree.

*E. tef* likely evolved from the wild allotetraploid *E. pilosa* [57]. The progenitors of *E. pilosa* are not known, however the allopolyploidization event is estimated to have occurred from 4 [36] up to 6.4 mya [54]. It would be tempting to speculate that the subclades seen in *E. tef* include paralogs from two distinct populations deriving from the very same LTR-RT family, having colonized the two genome counterparts of the *E. pilosa* genome. The hypothesis is that the ancient LTR-RT family evolved separately into the two contributing genomes of *E. pilosa*. In the allotetraploid *E. pilosa*, the two LTR-RT populations continued to evolve separately.

We analyzed the sequence data available for both clades. Clade 1 includes 21 paralogs: 15 and 6 in subclade A and subclade B, respectively (Additional files 6a, 7). Clade 2 includes 22 paralogs: 18 in subclade 1 and 4 in subclade 2, respectively (Additional files 6b, 8). Each paralog from subclade A was compared at the nucleotide level with all the paralogs in subclade B, separately for clades 1 and 2, in order to estimate the nucleotide distance separating each pair. The distances were translated into millions of years following the molecular paleontology strategy described by San Miguel et al. [58] using the substitution rate of 6.5 × 10^−8^ calculated for rice [29]. The insertion time estimates range from 9 to 32 mya and from 14 to 26 mya for clades 1 and 2, respectively. This limited evidence would seem to support the view that the two LTR-RT populations split well before the *E. pilosa* origin. However the lack of concrete data regarding the progenitors of *E. pilosa*, and the time of their separation from the common progenitor, as well as the unavailability of any extensive genome sequence data from all these species dramatically limit the possibility of further testing this hypothesis.

**Fig. 3:**
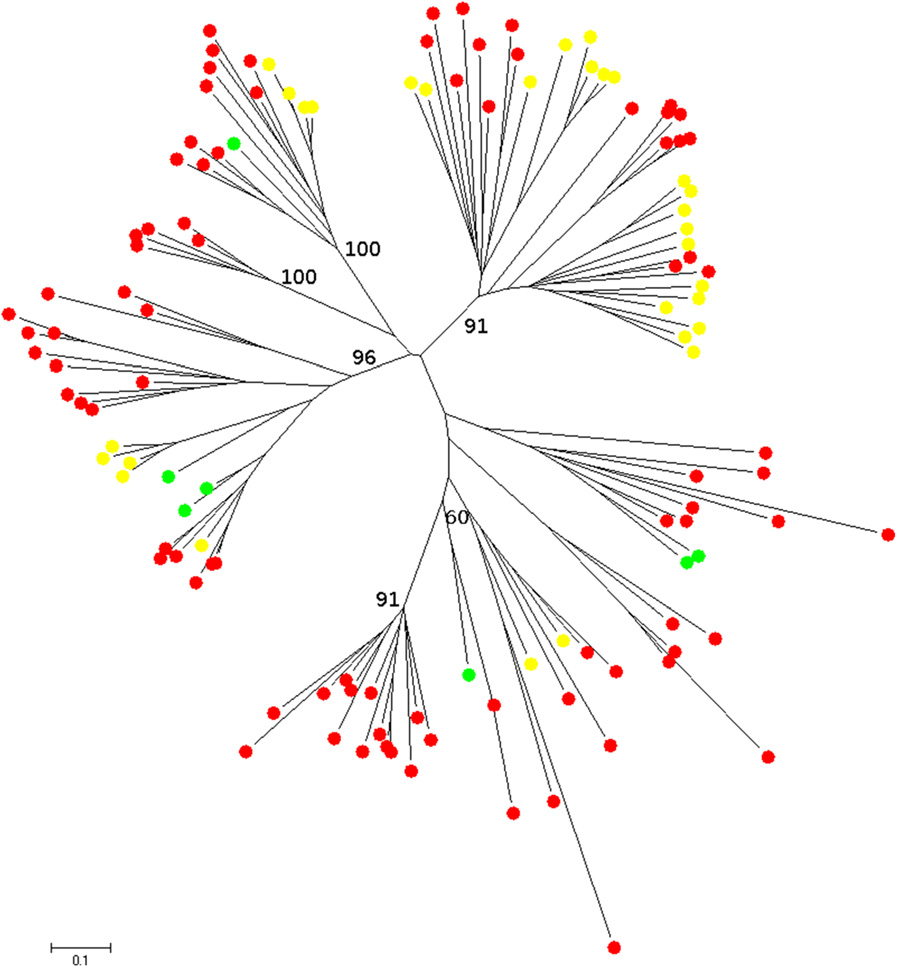
Phylogenetic analysis of non-LTR retroelements. Bootstrap values were calculated for 1000 replicates; only those greater than 50 are shown. Paralogs from maize elements are marked with yellow circles; those from rice with green circles, and those from teff with red circles

For non-LTR retroelements, 123 paralogs were identified and analyzed: 86 from *E. tef*, 7 from rice and 30 from maize. Roughly half of the teff paralogs mixed with those of rice and maize, reflecting the fact that most of these elements are ancient and are shared between the three species although a certain amount of proliferation occurring after speciation was detected (Fig. 3).

Phylogenetic analysis was then extended to three of the most representative groups of DNA TEs: CACTA, MuDR and hAT. Paralog tracts of the transposase domain of CACTA and MuDR elements and of the dimerization domain of hAT elements were identified in the three species analyzed. Paralogs were aligned (Additional files 9, 10 and 11 and then used to build NJ phylogenetic trees.

**Fig. 4:**
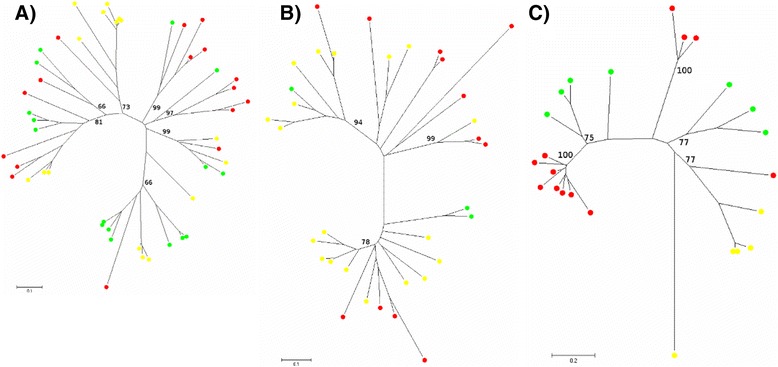
Phylogenetic analysis of DNA transposable elements. Bootstrap values were calculated for 1000 replicates; only those greater than 50 are shown. Paralogs from maize elements are marked with yellow circles; those from rice with green circles, and those from teff with red circles. **a**) CACTA; **b**) hAT; **c**) MuDR

The 48 CACTA paralogs (16 copies each for teff, rice and maize) and the 34 hAT-like ones (12 copies for teff, 19 for maize and 3 for rice) showed similar patterns (Fig. 4a and b) to those previously described for non-LTR retroelements (Fig. 3). Conversely, most of the 12 *E. tef* MuDR paralogs clustered separately in species-specific highly bootstrap-supported clades, thus suggesting a recent activity and differentiation of this group of TEs in teff (Fig. 4c).

**Fig. 5:**
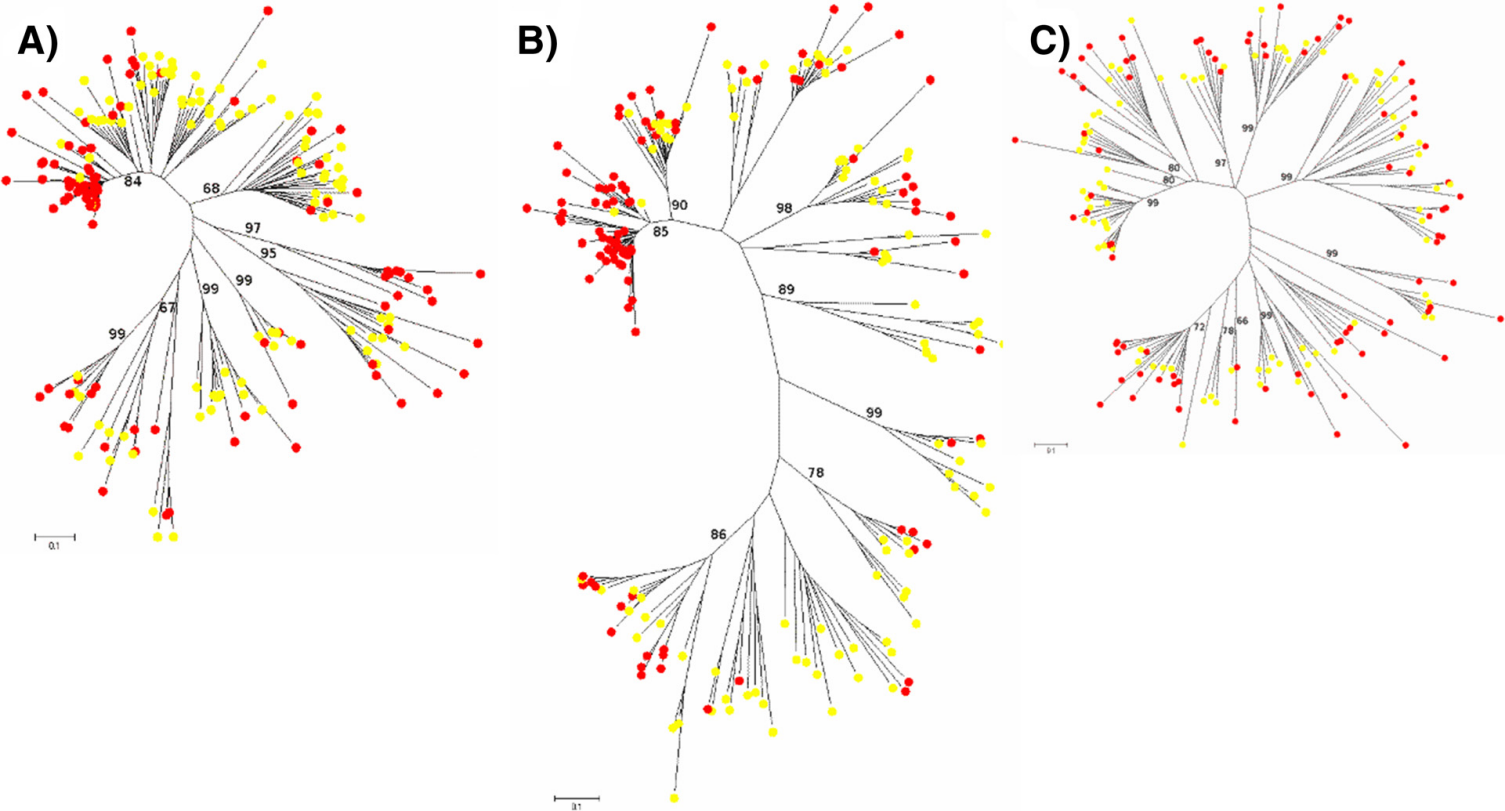
Phylogenetic analysis of retroelements in Enantite and Tsedey cultivars. Bootstrap values were calculated for 1000 replicates; only those greater than 50 are shown. Paralog elements from Tsedey cv are marked with yellow circles; and those from Enantite cv. with red circles. a) Ty1-copia; b) Ty3-gypsy; c) Non-LTR retroelements

We exploited a draft sequence from a different *E. tef* cultivar (Tsedey) to analyze the philogenetic relationships of Ty1-copia, Ty3-gypsy and non-LTR retro-elements in the two cultivars. For each of the three TE classes, from the total amount of identified paralog RT tracts we randomly retrieved 100 copies each for both Tsedey and Enantite cultivars. The sequences were aligned (Additional files 12, 13 and 14) and used to build NJ phylogenetic trees. For both Ty1-copia and Ty3-gypsy, the majority of paralogs mixed together suggesting that the activity leading to the production of extant copies mainly took place before the two cultivars separated (Fig. 5a and b). However some cultivar specific clades were identified, possibly indicating recent differential TE activity in the two cultivars. If these specific clades represent real evolutive events then a selective proliferation of certain LTR-RT families after cultivar selection should be assumed. In this case however, the paralogs would exhibit extremely short branches reflecting a recent and fast amplification. Since this does not seem to be the case, the most likely explanation is that the evidence is artifactual and possibly due to a selective sampling of few LTR-RT subpopulations in the assembled sequence (i.e. cultivar Tsedey). In the case of non-LTR retroelements, almost all the clades included paralogs from both cultivars (Fig. 5c).

### An abundant satellite sequence

A tandem-arranged satellite sequence was identified as one of the most abundant repeats in the *E. tef* genome. We mined the dataset of random sheared reads for representative monomers of this repeat. Out of the 250,000 reads searched, 26,595 positive hits were obtained using RepeatMasker [45]. One thousand of these hits, each representing a complete satellite monomer, were randomly extracted from the total and used for further analyses (Additional file 15). The length of the consensus monomer, as identified by Tandem Repeat Finder software [46], is 169 bp. The monomer length ranges from 163 to 177 bp. The average GC content is: 45.21 %. The consensus sequence of the monomer did not provide any significant hits when it was used to search the comprehensive database of plant satellite sequences plantSatDB [59]. The overall similarity among the 1,000 random copies was 79 %. However more than half the copies (554) had a greater than 94 % similarity with at least another copy in the random dataset. The variation in conservation across the monomer sequence was investigated by analyzing one thousand monomer copies to create a consensus-logo (Additional file 16). A consensus-logo is a graphical representation of the sequence where the height of each residue reflects its conservation in that position across the sequence copies analyzed [60]. Conservation is quite pronounced across the entire sequence. The estimated overall abundance across genomes (i.e. 4.54 %) assuming a genome size of 730 Mbp and an average length of the monomer of 169 bp, translates to a greater copy number than 196,000.

**Fig. 6:**
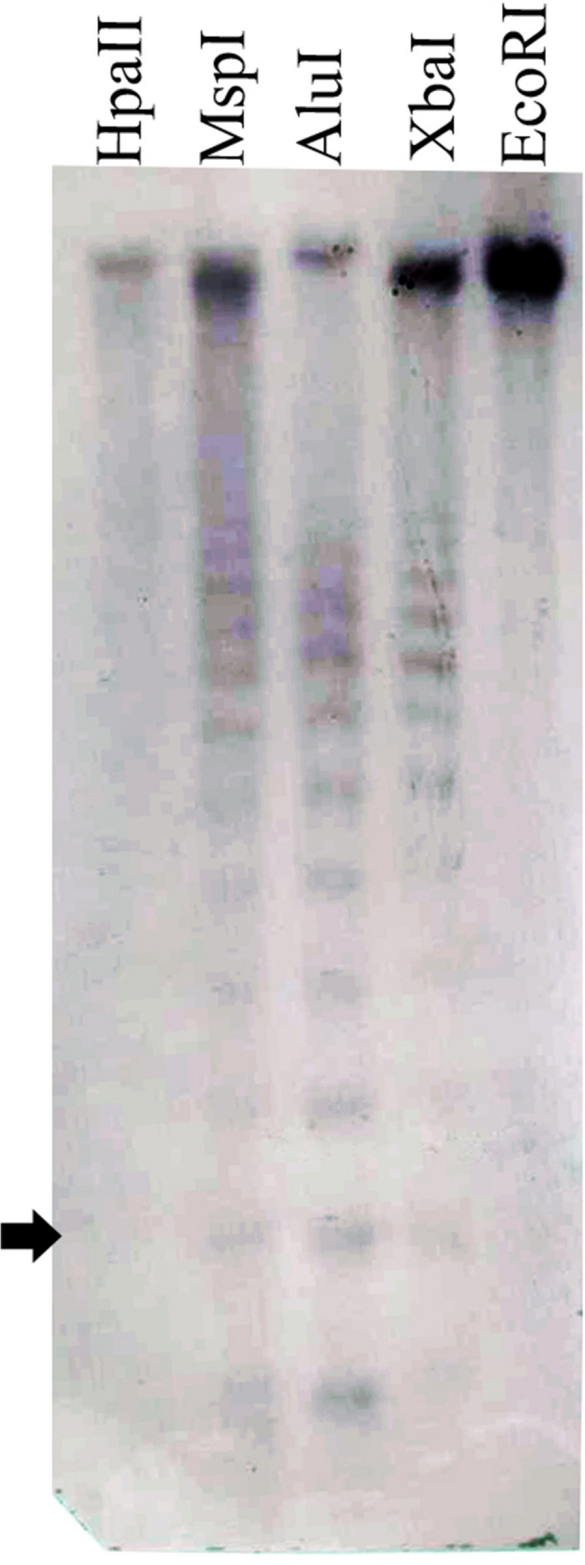
Southern Blot Hybridization of the Satellite repeat. The arrow indicate the band corresponding to monomer length (i.e. 165 bp)

Similarity searches also detected this sequence in the assembled scaffolds of teff cultivar Tsedey. As expected the overall amount of this sequence in scaffolds was extremely reduced (a few hundred copies) since the satellite rich regions of the genome are extremely difficult to assemble. However, a similarity search carried out on a random sample of raw illumina reads (from teff cv. Tsedey library GYN 7, SRR1463355) using the satellite sequence as a query masked 2.89 % nucleotides. This figure is consistent with the one calculated for cv. Enantite. To further examine the features of this satellite sequence, to confirm the evidence gained from in silico analysis and to rule out any possible artifactual finding due to library construction [61] or sequencing issues, a Southern blot hybridization experiment was carried out. Five different restriction enzymes were used. Four of them (*Xba*I, *Alu*I, *Msp*I, HpaII) recognize a restriction site inside the analyzed sequence, one does not: *Eco*RI. The signals produced by hybridization were quite strong confirming the fact that this sequence was abundant. Furthermore all the restriction enzymes (with the exception discussed later of *Hpa*II) having a restriction site in the satellite sequence gave rise to the expected “ladder-like” pattern, thus confirming the tandem arrangement of this sequence (Fig. 6). *Msp*I and *Hpa*II are two isoschyzomeres recognizing the sequence 5′-CCGG-3′. *Hpa*II is sensitive to the methylation of either of the two cytosines whereas *Msp*I is sensitive only to the methylation of the external one. The hybridization patterns for *Msp*I and *Hpa*II, showed major differences. In particular *Msp*I digest shows a clear ladder, *Hpa*II does not suggesting a higher degree of methylation of the internal cytosine in the target sequences. However both digests also showed an intense signal in the high molecular weight range suggesting some methylation of the external citosine. Taken together these results indicate a certain amount of methylation of this repetitive sequence.

## Discussion

The analysis of random sheared sequences assumed to represent an unbiased sample of the genome is a well established practice used to assess the repetitive content of genomes. This approach circumvents most of the limitations associated with the biased representation of repeats in whole genome assemblies [49, 62–64]. It is well known that repetitive sequences pose a serious technical challenge to genome assembly [65]. Along with misassemblies and gene misannotations [31], one of the most common and expected artifactual outcomes is an overall depletion of repeats in the final genome assembly, thus resulting in a severe underestimation of the overall amount of this class of sequences. For these reasons, in order to identify, analyze and characterize the genome component of *E. tef,* we analyzed a random subset of 500,000 reads covering about 0.25× of the whole genome by adopting a de novo strategy mostly by using RepeatScout [39]. We thus identified 1,389 putatively medium/highly repetitive sequences. We estimated that all of them mask more than 27 % of the genome. This value is much larger than the previous estimate of about 14 % repeat content in teff [36] based on the analysis of the available genome assembly.

Along with our strategy, we tested three other tools that exploit next generation sequence data: repArK, TEDNA and RepeatExplorer. The strategy we adopted outperformed two of these tools (RepArk and TEDna) and compared well with RepeatExplorer. However, irrespectively of the specific tool used, the de novo identification approach requires considerable effort in the accurate characterization of the repetitive candidates isolated. In particular, all the sequences that are repetitive by nature but not similar to TEs or to satellite repeats such as members of gene families, ribosomal sequences, low complexity sequences and plastidial contaminants need to be identified and removed. Another disadvantage is that most of the repeats identified are not complete, thus leading to a severe fragmentation of the consensus sequence [47].

Roughly one third of the repeats that we identified (442) are related to LTR-RTs that represent most of the TE fraction in the teff genome as is the case in several plants [66]. Altogether LTR-RTs were estimated to represent about 15 % of the teff genome. Considering similarly sized plant genomes, this value is comparable with that estimated in *Actinidia chinensis* (13.4 % out of 758 Mbp; [67]) and *Vitis vinifera* (14.32 % out of 487 Mbp; [68]) however it is much smaller than that calculated in tomato (62 % out of 460 Mb; [69]) and potato (53 % out of 311 Mb; [70]). As expected it is much smaller than the estimates in large genomes such as maize (> than 75 % out of 2,300 Mbp; [11]), barley (76 % out of 5,100 Mbp; [71]) and Norway spruce (about 60 % out of 20 Gbp; [72]).

Two possible reasons, amongst others, for the apparent underrepresentation of LTR-RTs in *E. tef* compared to similarly sized genomes are the presence of several highly diverged elements and/or an abundant population of single or low copy LTR-RTs. The two explanations are not mutually exclusive, however in both cases such elements would go undetected by de novo search [73]. The Ty3-gypsy superfamily appears to be much more abundant than Ty1-copia (11.40 % vs 2.67 %) as is the case in many plants such as the species of *Oryza* genus [9], maize [74] and *Brachypodium* [75]. We were unable to ascertain whether this unbalanced distribution was due to a different number of copies of the elements belonging to the two superfamilies or to a longer average length of Ty3-gypsy elements, because the repeats library used does not contain complete copies of LTR-RTs but only partial ones. However if the number of RT tracts identified is used as a proxy of the abundance of elements, the copia to gypsy ratio would be just 1:1.33, which is much less unbalanced than the value of 1:4 calculated using the amount of bases masked.

This suggests that the greater amount of gypsy elements could be explained not just in terms of the absolute copy number but also taking into account the longer length of these elements described in several plant genomes. For example, in rice Ty1-copia and Ty3-gypsy elements have an average length of 6.2 kb and 11.7 kb, respectively [76]. In cotton, the Ty3-gypsy average length is 9.7 Kbp, whereas for Ty-1 copia elements it is 5.3 Kbp [77, 78]. In flax (*Linus usatissimum*) Ty1-copia elements are on average 5.3 kb long and Ty3-gypsy are 8.7 Kbp [79]. Although no average values were provided for maize LTR-RTs when the twenty most abundant LTR-RT families were considered, Ty3-gypsy elements are often longer than Ty1-copia [74]. It is also possible that the presence of non-autonomous elements contributes to the excess of Ty3-gypsy. Other class I TEs were underrepresented: SINEs and non-LTR retrotransposoms represent just 0.18 % and 0.12 % of the genome, respectively. These results are consistent with the evidence gathered in many plant genomes [80]. Class II elements totaled 2.33 % of the teff genome, which is smaller than those estimated in many other cereal crops such as rice (12.96 %, [81]), *Brachypodium* (4.77 %, [75]), *Sorghum bicholor* (7.46 %, [10]) and maize (8.6 %, [11]). Most of the repeats library is composed of “uncharacterized repeats” (771), which could represent highly diverged TEs or scarcely conserved tracts of LTR-RTs such as the LTRs. All these regions obviously go undetected in similarity searches. In any case this large fraction of the library masked just 4.44 % of the genome. A previously undetected satellite like sequence was identified and partially characterized. It covered more than 4 % of the total genome size and its copy number was in the order of hundreds of thousands. The average length of the monomer, i.e. 169 bp, is close to the most common length of plant satellite sequences collected in PlantSatDB: 165 bp [59]. However, no significant similarity at the sequence level was detected with any of the entries in PlantSatDB. This is not surprising since these kinds of sequences show a great degree of variability even between closely related species [82, 83]. The high copy number, the length of the monomer and the tandem arrangement of this sequence suggest that it may play a role as a centromere component. However this conclusion cannot be reached solely on the basis of the data collected so far. Further studies and cytogenetic analyses are needed to better assess the satellite sequence distribution along the teff genome and to infer its structural and functional role. This satellite sequence, although depleted in the teff assembled scaffolds, was proved to be abundantly present in the teff Tsedey cultivar when raw sequences from this cultivar were analyzed.

We carried out an extensive study of the phylogenetic relationships between different TE classes in *E. tef*. A comparative approach was undertaken extending the analyses to two other grasses: rice and maize. In the case of the LTR-RT Ty1-copia elements, interesting evidence was found of the presence of various highly bootstrap-supported clades including elements from all the three species. Horizontal transfer (HT) could be the reason behind such close relatedness between paralog TE copies from species that diverged from each other various tens of millions of years ago. Indeed in the plant kingdom HT has been proved to be more common than previously thought [84]. An alternative but not mutually exclusive explanation is the more pronounced conservation of Ty1-*copia* elements over a long evolutionary timescale. In fact this has been proved for various Ty1-copia families, such as Angela/Martians [85] and Tvv1 [86] in angiosperms and PARTC in gymnosperms where the elements of this family showed a striking conservation over 200 million years of evolution [87].

On the other hand Ty3-gypsy paralogs mainly separated according to the different species in which they were isolated. This possibly reflects a lesser degree of conservation for this superfamily. However Ty1-copia paralogs show a greater heterogeneity than the Ty3-gypsy paralogs. In fact, in the Ty3-gypsy superfamily, more than half of the total amount of paralog sequences analyzed collapsed into a single clade. For both LTR-RT superfamilies, the phylogenetic analysis showed the existence of abundant teff specific clades including most of the Ty1-copia RT paralogs and the majority of the Ty3-gypsy paralogs. These findings suggest the presence of teff specific LTR-RT elements, mostly proliferating in recent evolutionary times, possibly post polyploidization (i.e. in the last 4–6.4 mya [36, 54]). This could be an effect of the “genomic shock” [88] triggered by polyploidization leading to teff speciation.

LTR-RT elements related to the abundant *Oryza* LTR-RT families Atlantys [55], RIRE2 [56] and RIRE1 [13] are scarcely represented in teff, thus demonstrating once again how closely-related elements could proliferate at strikingly different rates in different species [13, 78].

## Conclusions

Our in depth analysis of a random sheared sequence dataset from the teff cv. Enantite enabled us to obtain a comprehensive library including 1,389 medium/highly repetitive sequences representing more than 27 % of this genome. By exploiting whole genome shotgun sequence data to identify the repetitive component, our approach overcame the serious limitations of repeats depletion in genomes assembled de novo. Our results provide insight into TEs dynamics and evolutionary history in this species as well as details of the features of an abundant satellite sequence. We believe that our data represent a valuable resource for further analyses of the genome of this important orphan crop.

## Methods

### Plant Material and DNA Extraction

*Eragrostis tef* var Enatite (accession PI 524439; plantid Enatite) was acquired from USDA Agriculture Research Service Germplasm Resources Information Network (http://www.ars-grin.gov/npgs/). Seedlings from five plants grown in a growth chamber were collected after two weeks of planting, and ground by mortar and pestle using liquid nitrogen. Genomic DNA was extracted using the GenElute plant genomic DNA Miniprep (Sigma Aldrich). The final elution was performed with DEPC water instead of the protocol Elution solution. Isolated DNA was subjected to further phenolic purification and ethanol precipitation as per the standard procedures. Finally, quality was checked by using a spectrophotometer and electrophoresis at 1 % agrose gel. DNA samples were kept at −20 °C before being dispatched for sequencing.

### Library construction, DNA Quality check, Sequencing, and Assembly

Libraries were produced according to Nextera DNA sample preparation guide (Nextera DNA Sample Prep Kit 96 sample-ref 15028211) with the following modifications:

- Gel extraction after fragmentation of genomic DNA (fragments were selected in the range 300–700 bp) was performed using certified low range ultra agarase-BIO-RAD (catalog 161–3107);
- the fragmented DNA was cleaned up using a QIAquick gel extraction kit (cat.28704) Qiagen
- PCR amplification: 7 cycles were carried out instead of 5.

DNA quality control was performed using Agilent Technologies 2100 Bioanalyzer and a high sensitivity DNA chip.

Sequencing was carried out using a MiSeq reagent kit v3 (600 cycle) cat. MS-102-3003 Illumina. The reagents kit up to 625 cycles of sequencing on the MiSeq system includes paired-end reagent plate (600 cycle), MiSeq flow cell and wash buffer.

Two libraries of raw DNA sequence pair end reads sequenced by MiSeq platform (300 bp for each end) were merged using PEAR [89].

### Repeats identification

Two sets of 250,000 reads each (xaa and xab) were randomly selected out of the total amount of sequences merged by PEAR, and used for de novo repeats identification and characterization. The strategy used has three steps:

- RepeatScout [39], was run separately on the two sets using default parameters to identify any repetitive sequence longer than 100 bp, present in more than 10 copies and without low complexity.
- Since RepeatScout is tailored to work with assembled genomes or, at least with long sequences, it is expected that the output obtained by analyzing short reads will be highly fragmented. In order to further assemble, if possible, the repetitive candidates identified and to produce longer consensus sequences, the two outputs were processed separately using CAP3 [40] run under relaxed settings (−o 30-p 80-s 500).
- The repeat consensus sequences obtained from b) were then analyzed using cd-hit [41] to collapse together all the sequences sharing at least an 80 % similarity.

To test the effectiveness of the strategy in capturing the medium/highly repetitive fraction of the genome, the results were compared to those obtained using RepeatExplorer [49], TEDna [48] and RepArk [47] using their default settings.

RepeatExplorer was fed with a dataset of 1,000,000 PEAR assembled reads. The overall result included 42,045 sequences. Only two hundred clusters containing the most represented sequences (2,722) were used for further analyses (i.e. low copy number repeats were excluded).

RepArk was run on 500,000 sequences and produced an output of 1,019 repeat candidates.

TeDNA was used to analyze two batches of 250,000 reads, each providing an output containing altogether 306 repetitive candidates.

### Library characterization

The characterization of the repetitive sequences was carried out on the basis of the results of similarity searches and sequence structural features analysis. In particular:

- putative repetitive sequences were compared at both nucleotide and amino acid levels with all the plant sequences included in RebBase [51] using Blast [90] and setting an e-value of 1e-5 as a threshold to identify significant hits.
- The sequences that did not provide any significant hit were then compared against the nr division of GenBank [91] using Blast search tools under the same conditions stated in point a). Sequences having similarity with plastidial sequences (both mitochondria and chloroplast) or with known gene families were removed from the dataset. Sequences with significant hits with known TEs were annotated accordingly and sequences with no hits were flagged with the term “NHF” i.e. “No hits found”. The latter are repetitive sequences not yet fully characterized.
- The repetitive library was then further analyzed to identify any sequence containing tandem-arranged motifs with a repetitive monomer longer than 100 nt. This analysis was done using Tandem Repeat Finder [46].

### Phylogenetic analysis

Tracts of 100 amino acid residues from the reverse transcriptase (RT) domains of Ty1-*copia*, Ty3-*gypsy* and non-LTR retroelements and 100 aa residues long tracts of the transposase domain of CACTA and MuDR elements and the dimerization domain of hAT elements (Additional file 17), were used as queries in TblastN searches against the 250,000 reads dataset xaa.

All the matches with an *E*-value lower than 1e-05 and covering at least 80 aa of the query sequence were retained. Paralog sequences from the most abundant and representative LTR-RTs identified in rice and maize were retrieved from Repbase [51], RetrOryza [50] and MaizeTEDB (http://maizetedb.org/~maize/) and added to the teff dataset. All the paralogs were then aligned separately for each TE class using Muscle [92]. The multiple alignments were then used to build NJ trees using MEGA version 6 [93] and the bootstrap values obtained after 1,000 replicates were calculated.

LTR-RTs and non-LTR retroelements conserved RT tracts were also mined from the available genome assembly of the teff cultivar Tsedey [36] and then aligned along teff, cv. Enantite paralogs in order to build NJ trees.

Nucleotide distances were calculated using “distmat” from EMBOSS [94], applying the Kimura 2 parameters model [95].

### Sequence Logo

**The** logo for the satellite sequence was produced using Web-logo [60].

### Southern blot hybridization

DNA was extracted from *E. tef* var Enatite seedlings, grown as described in “Plant material and DNA extraction”. For each enzymatic reaction, 5 μg of DNA was individually digested with the following restriction endonucleases: *Xba*I (R0145S; New England BioLabs), *Eco*RI (R0101S; New England BioLabs), *Hpa*II (R0171S; New England BioLabs), *Msp*I (R0106S; New England BioLabs) and *Alu*I (R0137S; New England BioLabs) following the manufacturer’s protocol.

DNA probe was made by isolating the target satellite sequence using the PCR reaction and the primers Forward (5′-CGG-TTA-TTT-CTG-TGT-TGT-TTC-GG-3′) and Reverse (5′-TGA-CCA-GTC-TGC-AGC-AAA-AC-3′) which were specifically designed for this purpose. The expected amplified band was extracted and purified using Wizard SV Gel and PCR Clean-up System (Promega). It was then diluted in 1:200 and used for labeling reactions by polymerase chain reaction (PCR) using DIG-11-dUTP labeling (Roche).

The digests were run on 1.5 % agarose gel for 2 h with a cold 0.5× TBE buffer. The gel was then soaked with GelRed for 10 minutes in order to visualize the gel under UV light. DNA was transferred to the positively charged nylon membrane (Roche). An NBT/BCIP (DIG High Prime DNA Labeling and Detection Starter Kit I by Roche) colorimetric detection system was used to visualize the hybridization profiled on the membrane.

### Availability of data and materials

The raw sequence data used in this work were submitted to GenBank under the BioProject accession number PRJNA294641. The datasets relative to phylogenetic and sequence analyses supporting the conclusions of this research are included within the article and listed in the “additional files” section.

## Additional files

Additional file 1:
**Library Etef_repeats_V1.4 containing the repeats isolated in this study.** (FAS 615 kb) Additional file 2:
**a) Sequence of a repeat library entry including ~2.5 copies of a tandem-arranged monomer.** Arrows indicate single monomers b) Dot plot self-comparison of the repeat library entry (PNG 71 kb) Additional file 3:
**Multiple alignment of Ty1-copia RT paralog sequences identified in teff, Rice and Maize.** (MSF 110 kb) Additional file 4:
**Multiple alignment of Ty3-gypsy RT paralog sequences identified in teff, Rice and Maize.** (MSF 135 kb) Additional file 5:
**Multiple alignment of non-LTR RT paralog sequences identified in teff, Rice and Maize.** (MSF 33 kb) Additional file 6:
**Detail of the clades splitting into two subclades (1 and 2) presented in Fig.** **2** **.** Bootstrap values were calculated for 1000 replicates; only those greater than 50 are shown. (PDF 27 kb) Additional file 7:
**Nucleotide sequences from paralogs in Clade 1 (Additional file** **6** **: Figure S6).** (FAS 10 kb) Additional file 8:
**Nucleotide sequences from paralogs in Clade 2 (Additional file** **6** **: Figure S6).** (FAS 6 kb) Additional file 9:
**Multiple alignment of CACTA transposase paralog sequences identified in teff, Rice and Maize.** (MSF 12 kb) Additional file 10:
**Multiple alignment of hAT dimerization domain paralog sequences identified in teff, Rice and Maize.** (MSF 7 kb) Additional file 11:
**Multiple alignment of MuDr transposase paralog sequences identified in teff, Rice and Maize.** (MSF 7 kb) Additional file 12:
**Multiple alignment of Ty1-copia RT paralog sequences identified in teff cv Enantite and Tsedey.** (MSF 51 kb) Additional file 13:
**Multiple alignment of Ty3-gypsy RT paralog sequences identified in teff cv Enantite and Tsedey.** (MSF 49 kb) Additional file 14:
**Multiple alignment of non-LTR RT paralog sequences identified in teff cv Enantite and Tsedey.** (MSF 45 kb) Additional file 15:
**1,000 sequences of the satellite monomer.** (FAS 185 kb) Additional file 16:
**Sequence logo analysis of the satellite sequence.** (PNG 93 kb) Additional file 17:
**Tracts of TE coding domains used as queries in similarity searches to retrieve copies of TE paralogs.** (FAS 663 bytes)

## Abbreviations

aa: amino acid
Bp: base pair
LTR: long terminal repeat
LTR-RT: long terminal repeat retroelement
NJ: neighbor-joining
nt: nucleotide
RT: reverse transcriptase
TEs: transposable elements
