# Supplementary figures and images for: Identification and characterization of abundant repetitive sequences in Eragrostis tef cv. Enatite genome
Source: BMC Plant Biol. 2016 Feb 1;16:39. doi: 10.1186/s12870-016-0725-4 (PMC4736629; doi:10.1186/s12870-016-0725-4)

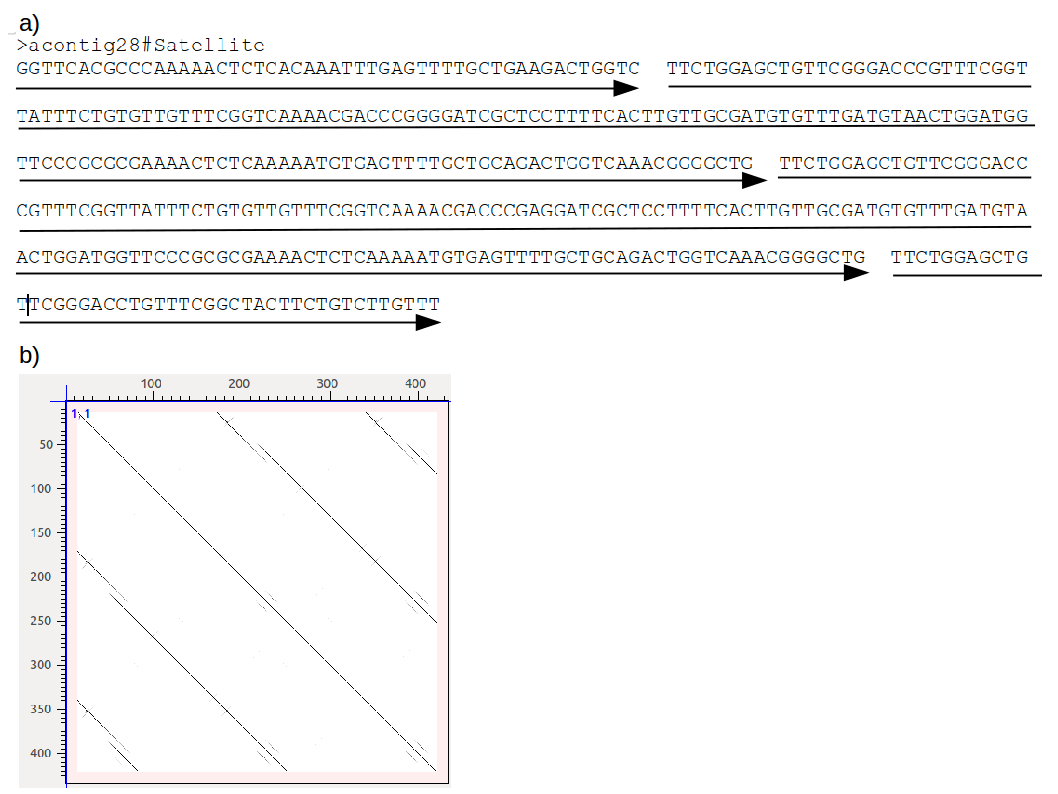

Supplement: Additional file 2: — a) Sequence of a repeat library entry including ~2.5 copies of a tandem-arranged monomer. Arrows indicate single monomers b) Dot plot self-comparison of the repeat library entry (PNG 71 kb) [file 12870_2016_725_MOESM2_ESM.png]

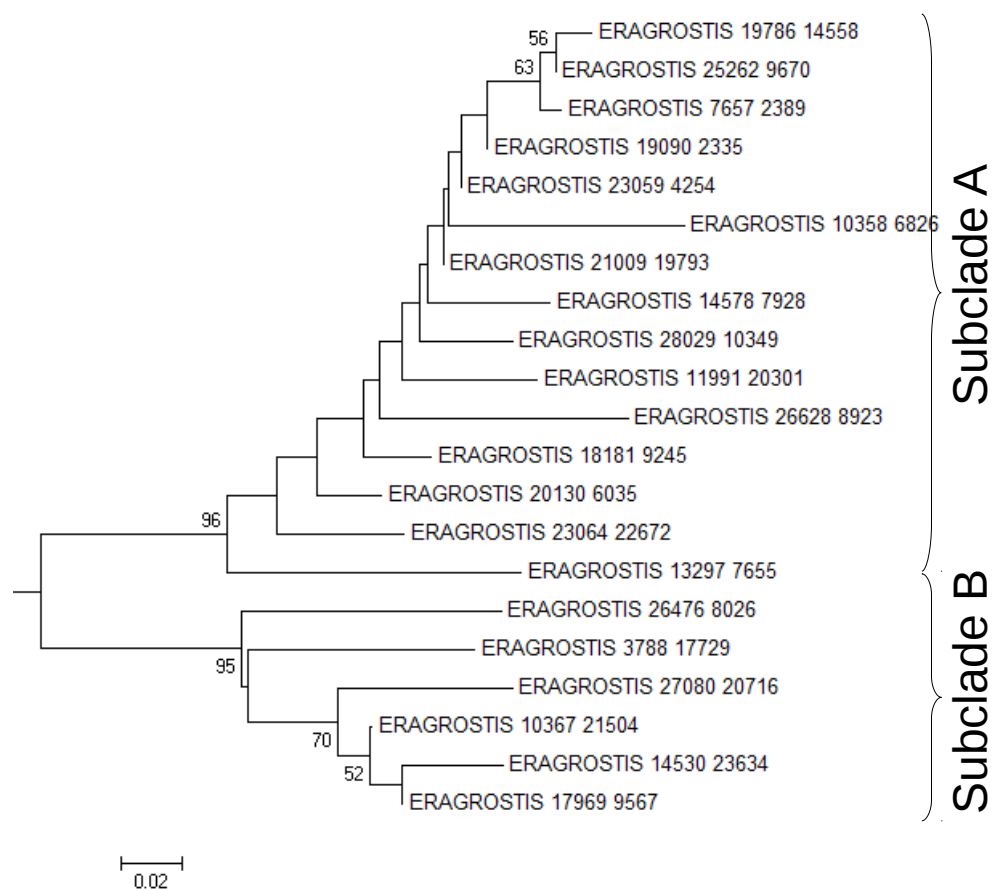

A

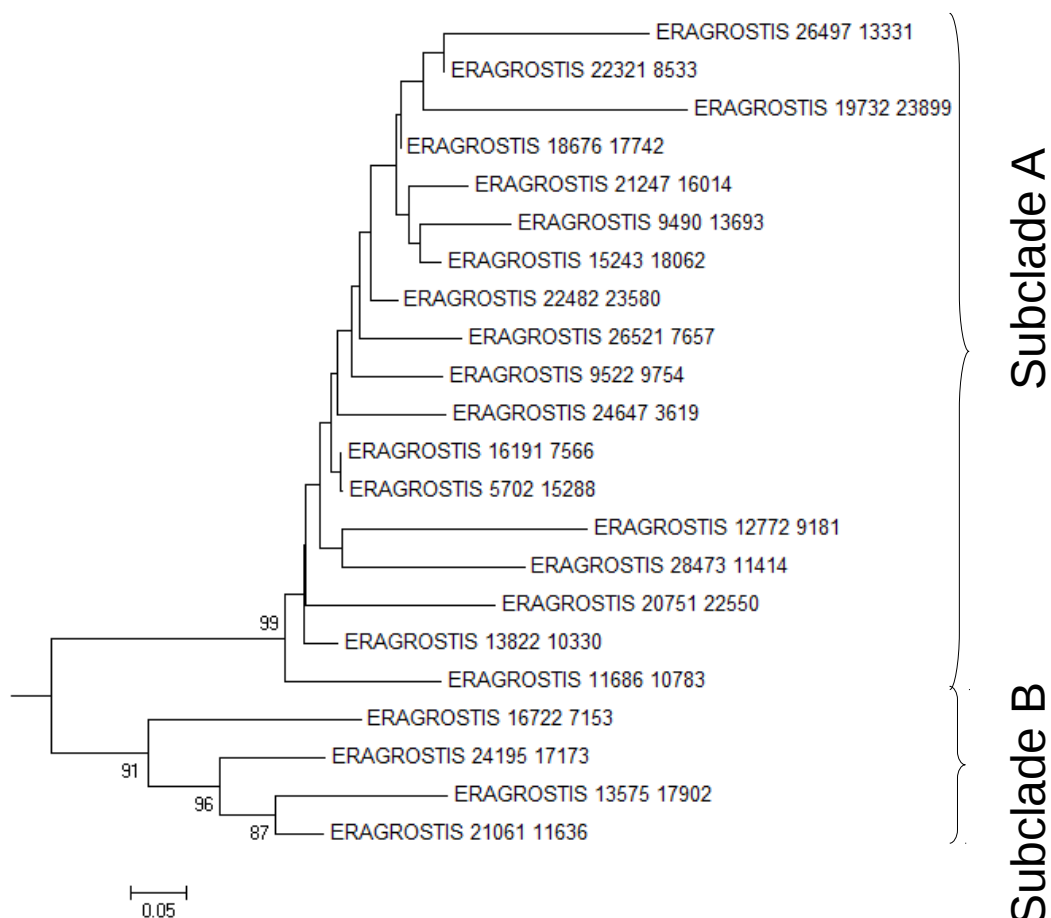

B

Supplement: Additional file 6: — Detail of the clades splitting into two subclades (1 and 2) presented in Fig. 2 . Bootstrap values were calculated for 1000 replicates; only those greater than 50 are shown. (PDF 27 kb) [file 12870_2016_725_MOESM6_ESM.pdf]

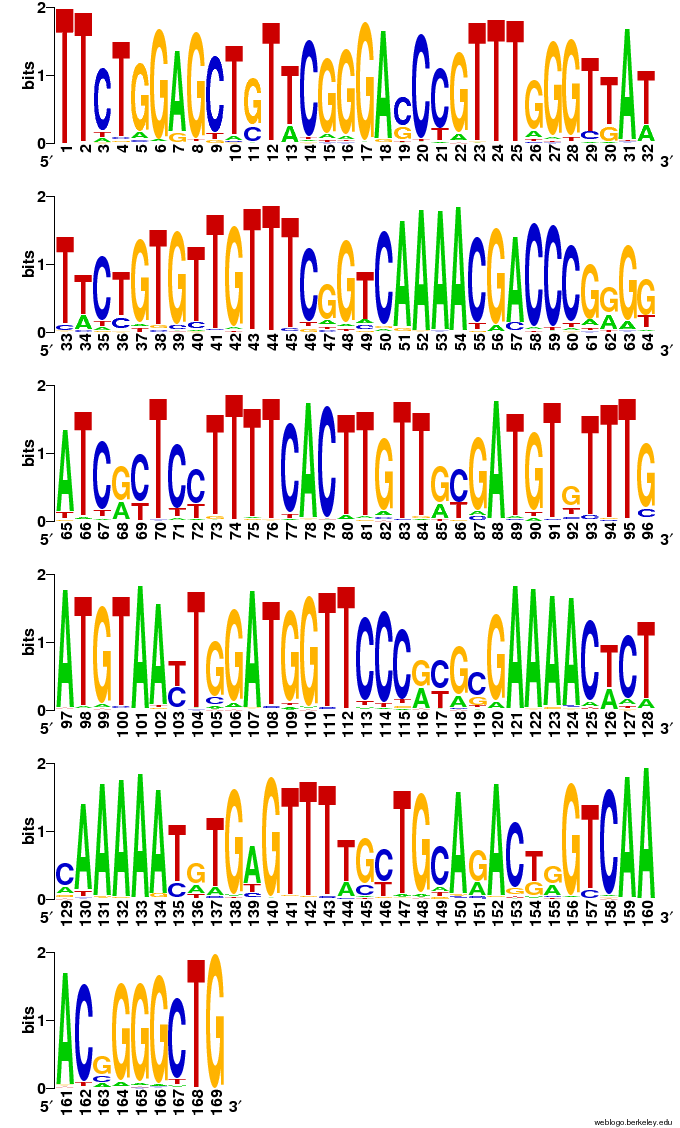

Supplement: Additional file 16: — Sequence logo analysis of the satellite sequence. (PNG 93 kb) [file 12870_2016_725_MOESM16_ESM.png]
